# Role of circulating polyunsaturated fatty acids on cardiovascular diseases risk: analysis using Mendelian randomization and fatty acid genetic association data from over 114,000 UK Biobank participants

**DOI:** 10.1186/s12916-022-02399-w

**Published:** 2022-06-13

**Authors:** Maria Carolina Borges, Philip C. Haycock, Jie Zheng, Gibran Hemani, Michael V. Holmes, George Davey Smith, Aroon D. Hingorani, Deborah A. Lawlor

**Affiliations:** 1 MRC Integrative Epidemiology Unit, University of Bristol, Oakfield House, Oakfield Grove, Bristol, BS8 2BN UK; 2 Population Health Sciences, Bristol Medical School, University of Bristol, Bristol, UK; 3 MRC Population Health Research Unit (MRC PHRU), Nuffield Department of Population Health, University of Oxford, Oxford, UK; 4 Institute of Cardiovascular Science, Faculty of Population Health Sciences, University College London, London, UK; 5 UCL BHF Research Accelerator, London, UK; 6 Health Data Research UK, Institute of Health Informatics, University College London, London, UK; 7 UCL NIHR Biomedical Research Centre, London, UK; 8 NIHR Bristol Biomedical Research Centre, Bristol, UK

**Keywords:** Fatty acids, Cardiovascular diseases, Mendelian randomization

## Abstract

**Background:**

Despite early interest in the health effects of polyunsaturated fatty acids (PUFA), there is still substantial controversy and uncertainty on the evidence linking PUFA to cardiovascular diseases (CVDs). We investigated the effect of plasma concentration of omega-3 PUFA (i.e. docosahexaenoic acid (DHA) and total omega-3 PUFA) and omega-6 PUFA (i.e. linoleic acid and total omega-6 PUFA) on the risk of CVDs using Mendelian randomization.

**Methods:**

We conducted the largest genome-wide association study (GWAS) of circulating PUFA to date including a sample of 114,999 individuals and incorporated these data in a two-sample Mendelian randomization framework to investigate the involvement of circulating PUFA on a wide range of CVDs in up to 1,153,768 individuals of European ancestry (i.e. coronary artery disease, ischemic stroke, haemorrhagic stroke, heart failure, atrial fibrillation, peripheral arterial disease, aortic aneurysm, venous thromboembolism and aortic valve stenosis).

**Results:**

GWAS identified between 46 and 64 SNPs for the four PUFA traits, explaining 4.8–7.9% of circulating PUFA variance and with mean F statistics >100. Higher genetically predicted DHA (and total omega-3 fatty acids) concentration was related to higher risk of some cardiovascular endpoints; however, these findings did not pass our criteria for multiple testing correction and were attenuated when accounting for LDL-cholesterol through multivariable Mendelian randomization or excluding SNPs in the vicinity of the *FADS* locus. Estimates for the relation between higher genetically predicted linoleic acid (and total omega-6) concentration were inconsistent across different cardiovascular endpoints and Mendelian randomization methods. There was weak evidence of higher genetically predicted linoleic acid being related to lower risk of ischemic stroke and peripheral artery disease when accounting by LDL-cholesterol.

**Conclusions:**

We have conducted the largest GWAS of circulating PUFA to date and the most comprehensive Mendelian randomization analyses. Overall, our Mendelian randomization findings do not support a protective role of circulating PUFA concentration on the risk of CVDs. However, horizontal pleiotropy via lipoprotein-related traits could be a key source of bias in our analyses.

**Supplementary Information:**

The online version contains supplementary material available at 10.1186/s12916-022-02399-w.

## Background

Early interest in the cardiovascular effects of polyunsaturated fatty acids (PUFA) emerged from observational studies conducted between the 1950s and 1970s indicating that populations with a high intake of omega-3 PUFA had lower rates of mortality from cardiovascular diseases (CVDs) [1, 2]. Numerous subsequent randomized controlled trials (RCTs) indicated that dietary substitution of carbohydrates or saturated fatty acids by PUFA had a protective effect on intermediate outcomes, such as a reduction in low-density lipoprotein (LDL)-cholesterol and triglycerides [3]. However, the hypothesized cardioprotective role for omega-3 and omega-6 PUFA has been challenged by a recent series of Cochrane’s systematic reviews of RCTs of dietary advice or supplementation, which suggest little to no benefit [4–6]. Overall, most RCTs on PUFA intake included in these systematic reviews were at moderate to high risk of bias and there is large uncertainty on the evidence linking PUFA to several cardiovascular outcomes [4–6]. Despite being the gold-standard study design for testing the effect of clinical interventions, in practice RCTs are often limited in statistical power, breadth of outcomes analyzed, and have high risk of bias. Therefore, integrating multiple lines of evidence is key to improve causal inference on the role of PUFAs in CVD aetiology.

Alpha-linolenic acid and linoleic acid are omega-3 and omega-6 PUFAs, respectively, that cannot be produced endogenously by humans and, therefore, need to be obtained from diet. Other omega-3 and omega-6 PUFAs can be produced endogenously through a series of elongation and desaturation reactions [7]. Circulating PUFA concentration is influenced by environmental and genetic factors [8–13], and the measurement of circulating PUFA can be used as an objective biomarker of PUFA intake, avoiding well-known biases in self-reported assessment of dietary intake. Comparing individuals genetically predisposed to higher or lower circulating PUFA can be used to probe the lifelong effect of circulating PUFA on CVD risk using Mendelian randomization [14]. Mendelian randomization uses genetic variants associated with biomarkers as instrumental variables to assess their effect on disease aetiology. This approach was developed to improve causal inference by taking advantage of unique properties of genetic variants: (i) germline genotypes remain unchanged throughout one’s life, (ii) the random allocation of parental alleles at meiosis reduces confounding by generating balanced groups and (iii) the unidirectional flow of biological information (from genotype to phenotype) avoids reverse causation [15–17]. In addition to indexing lifelong exposure to a biomarker of interest, genetic variants are subject to relatively little bias due to measurement error [18].

Previous Mendelian randomization studies have reported conflicting findings regarding the relationship between circulating PUFAs and CVDs risk [19–25]. Overall, shorter (e.g. α-linolenic acid and linoleic acid) and longer (e.g. arachidonic acid) chain PUFA have been associated with lower and higher risk of CVDs, respectively [19–25]. These seemingly contradictory findings have been largely attributed to the inclusion of genetic variants mapping to the *FADS* locus in the analyses, which contains the genes *FADS1* and *FADS2* encoding, respectively, the desaturases delta-5 (D5D) and delta-6 (D6D), key enzymes catalyzing rate-limiting steps in PUFA biosynthesis [22]. Genetic variants modulating the expression/activity of D5D and D6D will lead to changes in shorter- and longer-chain PUFAs in opposite directions, which likely explains such contrasting MR findings of lower vs higher CVD risk being associated with shorter- vs longer-chain PUFAs, respectively. In addition, these *FADS* variants are highly pleiotropic and associated with numerous non-fatty acid traits including triglycerides, low-density lipoprotein (LDL)-cholesterol and fasting glucose [22]. On the one hand, given the well-established link of *FADS1/2* with PUFA biosynthesis, single-nucleotide polymorphisms (SNPs) in the vicinity of the *FADS* locus can add to the evidence on the involvement of fatty acids in the development of cardiovascular diseases. On the other hand, the fact that SNPs nearby *FADS1/2* are highly pleiotropic and not specific for individual fatty acids or fatty acid classes complicate inferences on the causal role of circulating fatty acids on CVD risk from Mendelian randomization studies solely/predominantly relying on genetic variants within this locus.

Some of these limitations in current Mendelian randomization studies could be addressed by incorporating into the analyses numerous genetic variants modulating circulating fatty acids via different pathways. Consistency of findings across variants from multiple loci would increase confidence in the results and allow the use of Mendelian randomization methods that require multiple independent variants and are more robust to violations of the method’s assumptions. However, the modest size of current genetic association studies (GWAS) on circulating fatty acids (up to ~13,000) have only allowed the discovery of a small number of genetic variants strongly and independently associated with circulating PUFA [8, 9, 11–13].

The aims of this study were (a) conducting the largest GWAS on circulating PUFA to date including a sample of 114,999 individuals and (b) using two-sample Mendelian randomization to investigate the involvement of circulating omega-3 and omega-6 fatty acids on a wide range of cardiovascular disease endpoints in up to 1,153,768 individuals of European ancestry (i.e. coronary artery disease, ischemic stroke, haemorrhagic stroke, heart failure, atrial fibrillation, peripheral arterial disease, aortic aneurysm, venous thromboembolism and aortic valve stenosis).

## Methods

### Data sources

#### Fatty acids

The UK Biobank is a population-based cohort of approximately 500,000 (~5% of those invited) people aged 40 to 69 years when recruited during 2006–2010 from several centres across the UK. A subset of approximately 20,000 were selected for repeat assessment between 2012 and 2013 [26, 27]. Details on study design, participants and quality control methods have been described previously [28, 29]. The UK Biobank received ethical approval from the Research Ethics Committee (REC reference for the UK Biobank is 11/NW/0382). This work was carried out using the UK Biobank project 30418 and 15825.

Circulating omega-3 (i.e. docosahexaenoic acid (DHA) and total omega-3) and omega-6 (i.e. linoleic acid and total omega-6) fatty acid concentration were measured using a targeted high-throughput nuclear magnetic resonance (NMR) metabolomics platform (Nightingale Health Ltd; biomarker quantification version 2020) [30]. Pre-release data from a random subset of 126,846 non-fasting plasma samples collected at baseline or first repeat assessment were made available to early access analysts. In total, 121,577 samples were retained for analyses after removing duplicates and observations not passing quality control (QC) (i.e. sample QC flag “Low protein”, biomarker QC flag “Technical error” or samples with insufficient material). This NMR platform provides simultaneous quantification of 249 metabolic measures (i.e. 165 metabolic measures and 84 derived ratios), encompassing routine lipids, lipoprotein subclass profiling (including lipid composition within 14 subclasses), fatty acid composition and various low-molecular weight metabolites such as amino acids, ketone bodies and glycolysis metabolites (Additional file 1: Table S1). Technical details and epidemiological applications of this platform have been previously reviewed [31–33]. The mean concentration of circulating fatty acids among the UK Biobank participants was 0.23 mmol/L (SD 0.08), 0.53 mmol/L (SD 0.22), 3.41 mmol/L (SD 0.69) and 4.45 mmol/L (SD 0.68) for DHA, total omega-3, linoleic acid and total omega-6, corresponding to 2%, 4.4%, 29% and 38% of total fatty acids, respectively.

#### Cardiovascular disease endpoints

The outcomes of interest were (prevalent/incident) coronary artery disease, ischemic stroke, haemorrhagic stroke, heart failure, atrial fibrillation, peripheral arterial disease, aortic aneurysm, venous thromboembolism and aortic valve stenosis. Table S2 (Additional file 1) describes the data sources used for these disease endpoints.

These data sources included several large-scale genetic consortia of cardiovascular disease outcomes [34–38] targeting individuals of European ancestry only, or predominantly, including the Coronary Artery Disease Genome-Wide Replication and Meta-analysis Plus the Coronary Artery Disease Genetics Consortium (CARDIoGRAMplusC4D) (*N* cases = 60,801; *N* controls = 123,504) [34, 39], MEGASTROKE (*N* cases = 40,585; *N* controls = 406,111) [35, 40], The Heart Failure Molecular Epidemiology for Therapeutic Targets (HERMES) (*N* cases = 47,309; *N* controls = 930,014) [38, 41], an atrial fibrillation genetic association meta-analysis (*N* cases = 60,620; *N* controls = 970,216) [36, 42] and an abdominal aortic aneurysm genetic association meta-analysis (*N* cases = 4,972; *N* controls = 99,858) [37].

In addition to consortia data, we used data from two large biobanks (i.e. the UK Biobank [26, 27] and FinnGen [43]). We selected 464,708 UK Biobank participants having European genetic ancestry, as defined by an in-house k-means cluster analysis performed using the first 4 principal components provided by the UK Biobank in the statistical software environment R [29]. We used a linear mixed model (LMM) association method as implemented in BOLT-LMM (v2.3) [44] to generate genetic association data on cardiovascular endpoints among the UK Biobank participants of European genetic ancestry as described previously [29, 45]. BOLT-LMM association statistics are on the linear scale; therefore, test statistics for these binary traits (betas and their corresponding standard errors) were transformed to log odds ratios and their corresponding 95% confidence intervals on the liability scale using a Taylor transformation expansion series [44]. FinnGen is a public-private partnership project combining genotype data from Finnish biobanks and digital health record data from Finnish health registries (https://www.finngen.fi/en). We used FinnGen data from release 4, which includes 176,899 participants [43]. The procedures used to generate genetic association data for cardiovascular outcomes in FinnGen have been described previously [43].

### Data analysis

#### Genome-wide association scan on circulating PUFA

We generated genome-wide genetic association data for circulating DHA, total omega-3, linoleic acid and total omega-6 concentration for 114,999 UK Biobank participants of European ancestry using BOLT-LMM (v2.3) [44, 46]. To model population structure in the sample, we used 143,006 directly genotyped SNPs, obtained after filtering on minor allele frequency (MAF) > 0.01; genotyping rate > 0.015; Hardy-Weinberg equilibrium *p*-value < 0.0001 and linkage disequilibrium (LD) pruning to an *r*^2^ threshold of 0.1 using PLINKv2.00. Genotype array, fasting time and sex were adjusted for in the model. All fatty acid measures were standardized and normalized prior to analyses using rank-based inverse normal transformation. SNPs with MAF < 1% or imputation accuracy (INFO) < 0.8 were excluded from further analyses. The same procedures were used to generate genome-wide genetic association data for other lipid-related traits measured using the NMR metabolomics platform [i.e. total fatty acids, triglycerides, clinical LDL-cholesterol (defined to match the LDL-C levels from routine clinical chemistry) and apolipoprotein B], which were used in follow-up analyses as detailed below. The genetic association data on fatty acids and other NMR traits were deposited at the IEU Open GWAS Project [47, 48].

We used LD score regression (LDSC) to estimate genome-wide inflation in test statistics from PUFA genetic association data due to population phenomena (e.g. population stratification), as well as to approximate the genetic correlation between circulating PUFA and between PUFA and other lipid-related traits (i.e. total fatty acids, LDL-cholesterol, triglycerides and apolipoprotein B) [49, 50].

#### Functional mapping and annotation of PUFA genetic association results

We used FUMA GWAS (“Functional Mapping and Annotation of Genome-Wide Association Studies”), an integrative web-based platform (http://fuma.ctglab.nl) containing information from 18 biological data repositories and tools, to characterize SNPs according to (i) consequences to gene function, (ii) mapped genes and biological pathways and (ii) associations with other phenotypes. The pipeline used by FUMA has been described in detail elsewhere [51]. Briefly, we applied FUMA’s SNP2GENE function, which uses genome-wide genetic association data to identify independent significant SNPs and SNPs in LD with those, which are then annotated for functional consequences to gene functions (i.e. altering expression of a gene, affecting a binding site or violating the protein structure) [52].

FUMA identifies as independent significant SNPs those associated with the trait of interested at *p*-value < 5 × 10^−8^ in the GWAS summary data and not in strong LD with each other (*R*^2^ < 0.6 using EUR 1000G phase3 as reference panel). For each independent significant SNP, all known SNPs with MAF ≥ 1% in strong LD (*R*^2^ ≥ 0.6), either present in the GWAS summary data and/or reference panel, are included for further annotation (i.e. candidate SNPs). Additionally, FUMA classifies independent lead SNPs as a subset of independent significant SNPs in weak LD (R^2^ < 0.1) and defines genomic risk loci by merging independent significant SNPs in LD blocks and between LD blocks closely located to each other (< 250 kb based on the most right and left SNPs from each LD block).

Functionally annotated SNPs are subsequently mapped to genes based on (i) physical position on the genome (positional mapping), (ii) expression quantitative trait loci (eQTL) associations (eQTL mapping) and (iii) 3D chromatin interactions (chromatin interaction mapping). In addition, independent significant SNPs and correlated SNPs are also linked to the GWAS catalog [53] to provide insights into previously reported associations of the SNPs with a variety of phenotypes. Gene-based test/gene-set analyses using MAGMA [54] are also carried out to summarize SNP associations at the gene level and associate the set of genes to biological pathways.

#### Selection of genetic instruments for PUFA

For our main Mendelian randomization analyses, we selected genetic variants strongly associated with circulating DHA, total omega-3 fatty acids, linoleic acid and total omega-6 fatty acids (*P*-value < 5 × 10^−8^) as instruments for these fatty acid measures using data from 114,999 UK Biobank participants. We performed pruning to remove variants in LD (*R*^2^ < 0.001 1000G EUR population) given we used Mendelian randomization methods that assume independence between genetic instruments. For each fatty acid measure, we approximated the total *R*^2^ and mean F statistics across selected SNPs, as previously described [55, 56]**,** using PUFA genetic association estimates from our discovery sample (i.e. the UK Biobank) and from a replication sample (Kettunen et al. [12]) to minimize bias due to winner’s curse. The replication sample corresponds to the largest previous GWAS (median *N* for fatty acids 13,516) with data on circulating fatty acids measured using the same NMR metabolomics platform as in the UK Biobank participants.

#### Assessing the impact of genetic instruments on the fatty acid pool

To explore the specificity of the genetic instruments for individual fatty acids, we assessed their impact on the composition of circulating PUFA using data from previous GWAS in individuals of European ancestry [8, 9, 11, 12], which have modest sample size (*N* range 7824–13,516) but more detailed data on individual PUFAs. These GWAS have been conducted in plasma samples using different assays (i.e. gas chromatography (GC), NMR or mass spectrometry (MS)) [8, 9, 11, 12] and have expressed results in standardized molar concentration units [12], as a proportion of total fatty acids [8, 9] or in arbitrary units [11]. We used the inverse variance weighted (IVW) method to test the impact of genetically predicted DHA, total omega-3, linoleic acid and total omega-6 on individual PUFA before and after excluding SNPs within 500 kb of the *FADS* locus (chromosome 11: 61,067,097 to 62,134,826).

#### Multivariable regression

For comparison with Mendelian randomization results, we fitted logistic regression models to estimate the association of circulating PUFA on the risk of each cardiovascular disease endpoint using individual-level data from the UK Biobank participants, with adjustments for covariables assessed at recruitment (i.e. sex, age, body mass index (BMI), fasting time, alcohol intake, frequency, statins use and total circulating fatty acids).

#### Mendelian randomization

We estimated the effect of genetically predicted DHA, total omega-3, linoleic acid and total omega-6 on the risk of each cardiovascular disease endpoint using two univariable summary data Mendelian randomization methods: IVW (with multiplicative random effects) and MR-Egger [57, 58]. IVW assumes no (unbalanced) horizontal pleiotropy. There are a range of plausible circumstances where this assumption could be violated, such as in the presence of pleiotropic SNPs that influence the outcome through pathways that are not mediated by the exposure (aka horizontal pleiotropy). Because of that, we also used the MR-Egger method, which can provide valid tests of a causal effect in the presence of unbalanced horizontal pleiotropy provided that instrument strength is independent of its direct effects on the outcome (i.e. INSIDE assumption). We used Cochrane’s Q and the MR-Egger intercept test to explore the presence of between-SNP heterogeneity and unbalanced pleiotropy, respectively, and leave-one-out analyses to test for the presence of outlying SNPs.

Given most circulating fatty acids are carried in the circulation by lipoproteins, we anticipated that many SNPs influencing circulating fatty acid concentration would map to genes regulating lipoprotein metabolism [12]. To mitigate bias due to lipoprotein-related traits, we used multivariable IVW to estimate direct effects of genetically predicted PUFA on the risk of cardiovascular disease endpoints after individually adjusting for total fatty acids, triglycerides, LDL-cholesterol, or apolipoprotein B, or simultaneously adjusting for both triglycerides and LDL-cholesterol. Multivariable IVW requires knowledge of the covariance between the effects of the genetic variants on each exposure, which we approximated from the phenotypic covariance matrix across exposure traits [59].

If genetic association data on cardiovascular diseases was available from two or more independent datasets (e.g. the UK Biobank and a European GWAS consortia not including the UK Biobank), we pooled these estimates using fixed-effect meta-analysis with inverse variance weights and conducted the MR analyses outlined above in both study-specific and pooled data. We used a Bonferroni correction to account for multiple testing considering the 9 different outcomes of interest (*p*-value = 0.05/9 outcomes = 0.006). Mendelian randomization analyses were performed using the TwoSampleMR [60] and MVMR [59] packages in R software version 3.6.2 (R Foundation for Statistical Computing).

#### Positive exposure control

For comparison with our Mendelian randomization analyses on circulating fatty acids, we used a positive control exposure approach, which consisted of selecting two exposures with well-established involvement in the risk of several cardiovascular diseases (i.e. LDL-cholesterol and apolipoprotein B) and conducting IVW to estimate their effect on the risk of the cardiovascular disease outcomes included in this study. Following the suggestion from a reviewer, we repeated these analyses after removing a SNP within the *FADS* locus.

## Results

### Genome-wide association scan on circulating PUFA

Genetic association results were available for 114,999 individuals and 12,321,876 genetic variants. FUMA identified 38, 41, 52 and 59 independent genomic regions strongly associated with circulating DHA, total omega-3, linoleic acid and total omega-6, respectively (Additional file 2: Fig. S1) [51, 52]. The Manhattan plots indicate a similar genetic association signature across PUFA, especially between DHA and total omega-3 and between linoleic acid and total omega-6 (Additional file 2: Fig. S2).

The quantile-quantile (QQ) plots did not indicate early separation of expected from observed *P*-values (Additional file 2: Fig. S3) and the LDSC intercepts did not indicate inflation of genome-wide test statistics due to confounding: 1.027 (SE = 0.010), 1.037 (SE = 0.010), 1.023 (SE = 0.011) and 1.029 (SE = 0.011) for DHA, total omega-3, linoleic acid and total omega-6, respectively.

As expected, genetic correlation was high between DHA and total omega-3 (0.85 (SE = 0.02)) and between linoleic acid and total omega-6 (0.95 (SE = 0.03)) and moderate to high between omega-3 and omega-6 fatty acids (ranging from 0.39 (SE = 0.07) between DHA and linoleic acid to 0.68 (SE = 0.06) between total omega-3 and total omega-6) (Additional file 1: Table S3). Overall, genetic correlations of circulating PUFA with other NMR lipid-related traits were null to moderate for DHA (ranging from −0.01 (SE = 0.08) for triglycerides to 0.57 (SE = 0.08) for LDL-cholesterol), but moderate to high for the other PUFA measures (ranging from 0.49 (SE = 0.07) between total omega-3 and triglycerides to 0.90 (SE = 0.02) between total omega-6 and total fatty acids) (Additional file 1: Table S3).

### Functional mapping and annotation of fatty acid genetic association results

Most genetic variants associated with circulating PUFA were either intronic or intergenic, suggesting that they are likely associated with PUFA concentration by modulating gene expression (Additional file 2: Fig. S1). As expected, genes mapped to PUFA-associated genetic variants included some genes with well-known role in lipoprotein metabolism, such as *CETP*, *PCSK9*, *LPL*, *LIPC*, *ANGPTL3*, *APOB*, *APOC3*, *APOA1*, *APOE* and *LDLR*, among others (Additional file 1: Tables S4A-D).

Using the full distribution of SNPs *p*-values, gene sets assigned by MAGMA were mostly related to pathways involved in lipoprotein metabolism. As an example, the top five results across PUFA measures were related to (triglyceride-rich or high-density) lipoprotein particle remodelling, apolipoprotein binding and reverse cholesterol transport (Additional file 1: Table S5A-D).

Using data from the GWAS catalog, we identified SNPs that associated with PUFA (or SNPs in strong LD with these) as correlated with numerous traits (Additional file 1: Table S6A-D). In particular, among the top ten results across PUFA were triglycerides, LDL-cholesterol, HDL-cholesterol, total cholesterol and C-reactive protein.

### Selection of genetic instruments for PUFA

The number of genetic instruments selected (*P*-value < 5×10^−8^; *R*^2^ < 0.001) using data from the UK Biobank participants was 46, 49, 55 and 64 SNPs for DHA, total omega-3, linoleic acid and total omega-6, respectively (listed in Additional file 1: Table S7). When combining information from SNPs present in both discovery and replication samples, the variance explained by these SNPs in the corresponding fatty acid measure was 6.5% (DHA), 7.9% (total omega-3), 4.8% (linoleic acid) and 5.2% (total omega-6) in the UK Biobank (discovery sample) in contrast to 3.1% (DHA), 3.7% (total omega-3), 6.9% (linoleic acid) and 5.7% (total omega-6) in Kettunen et al. data [12] (replication sample). The mean F statistics across SNPs ranged from 109 to 201 among fatty acid measures in the UK Biobank and from 9 to 18 in the Kettunen et al. data (Additional file 1: Table S8). If excluding SNPs nearby the *FADS* locus, the variance explained by these SNPs in the corresponding fatty acid measure in the UK Biobank was 3.1% (DHA), 4.5% (total omega-3), 4.5% (linoleic acid) and 5.2% (total omega-6).

### Assessing the impact of genetic instruments on the fatty acid pool

**Fig. 1:**
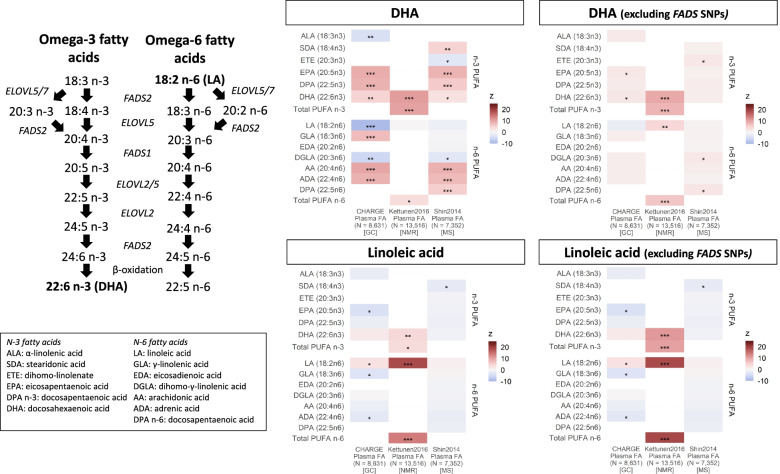
Association of genetically predicted DHA and linoleic acid on the circulating PUFA composition among individuals of European ancestry before and after excluding SNPs nearby the *FADS* locus. Results are expressed as *z*-statistics (i.e. effect estimate / standard error) for the variation in individual omega-3 and omega-6 fatty acids (*y*-axis) across multiple data sources (*x*-axis) according to genetically predicted DHA and linoleic acid. These are expressed using different shades of colours to indicate relative strengths of association; blue, red and grey boxes denote, respectively, decreases, increases and no change in individual PUFA, while white boxes represent missing data. Asterisks indicate *P*-value: < 5×10^−8^ (***), < 5×10^−5^ (**) and < 5×10^−2^ (*). DHA: docosahexaenoic acid; *FADS*: fatty acid desaturase genes; *ELOVL*: elongase genes; SNP: single-nucleotide polymorphism; Plasma FA: plasma fatty acids; N: median sample size used for estimating SNP-fatty acid association; GC: gas chromatography; NMR: nuclear magnetic resonance; MS: mass spectrometry

Genetic instruments predicting higher omega-3 fatty acids (i.e. DHA and total omega-3), selected from the UK Biobank participants, were associated with multiple individual fatty acids across all three independent datasets. Overall, higher genetically predicted DHA concentrations were related to higher longer chain (e.g. DHA and arachidonic acid) and lower shorter chain (e.g. α-linolenic acid and linoleic acid) PUFA (Fig. 1). After excluding SNPs nearby the *FADS* locus, genetically predicted DHA were no longer associated with most PUFA, and it became positively associated with linoleic acid (Fig. 1). Similar results were observed for total omega-3 fatty acids (Additional file 2: Fig. S4).

Genetic instruments predicting higher linoleic acid were strongly associated with increases in linoleic acid and omega-6 fatty acids, as well as DHA, in one out of the three independent datasets (Fig. 1). The exclusion of *FADS* SNPs had a modest impact on the relation between genetically predicted linoleic acid with PUFA composition (Fig. 1). Similar results were observed for total omega-6 fatty acids (Additional file 2: Fig. S4).

### Multivariable regression

Characteristics of the UK Biobank participants included in multivariable regression models are summarized in Table S9 (Additional file 1).

#### Omega-3 PUFA (DHA and total omega-3 fatty acids)

**Fig. 2:**
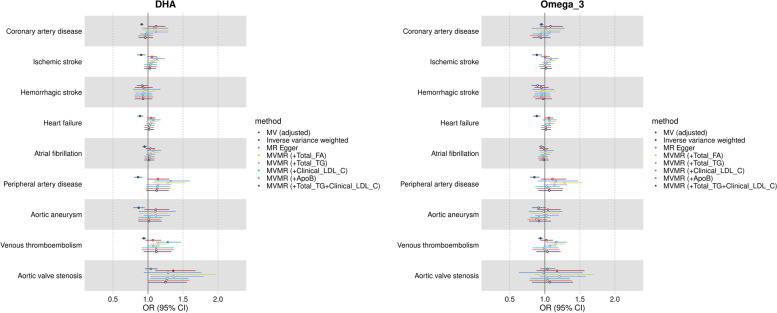
Multivariable regression and Mendelian randomization results for the risk of cardiovascular diseases associated with higher genetically predicted DHA and total omega-3 fatty acids among individuals of European ancestry. Results are expressed as odds ratio (OR) of cardiovascular diseases per standard unit increase in DHA and total omega-3 fatty acids with corresponding 95% confidence interval (CI). Full symbols indicate associations at *P*-value lower than the threshold accounting multiple testing (*P* < 0.006). Multivariable regression results are adjusted by for sex, age, BMI, fasting time, alcohol intake, frequency, statins use and total circulating fatty acids. DHA: docosahexaenoic acid; MV: multivariable regression; MR: Mendelian randomization; MVMR: multivariable Mendelian randomization; FA: fatty acids; TG: triglycerides; LDL: low-density lipoprotein cholesterol; ApoB: apolipoprotein B

In logistic regression models, higher circulating DHA was related to lower risk of most cardiovascular disease endpoints. The odds ratio of disease per standard unit higher DHA concentration (in models adjusted for sex, age, BMI, fasting time, alcohol intake frequency, smoking, statins use and total circulating fatty acids) was 0.91 (95% CI 0.89–0.94) for coronary artery disease, 0.90 (95% CI 0.84–0.97) for ischemic stroke, 0.92 (95% CI 0.84–1.00) for haemorrhagic stroke, 0.89 (95% CI 0.84–0.93) for heart failure, 0.95 (95% CI 0.92–0.98) for atrial fibrillation, 0.86 (95% CI 0.80–0.92) for peripheral artery disease, 0.87 (95% CI 0.79–0.95) for aortic aneurysm, 0.94 (95% CI 0.91–0.97) for venous thromboembolism and 1.04 (95% CI 0.95–1.14) for aortic valve stenosis. Similar results were observed for total omega-3 fatty acids (Fig. 2).

#### Omega-6 PUFA (linoleic acid and total omega-6 fatty acids)

**Fig. 3:**
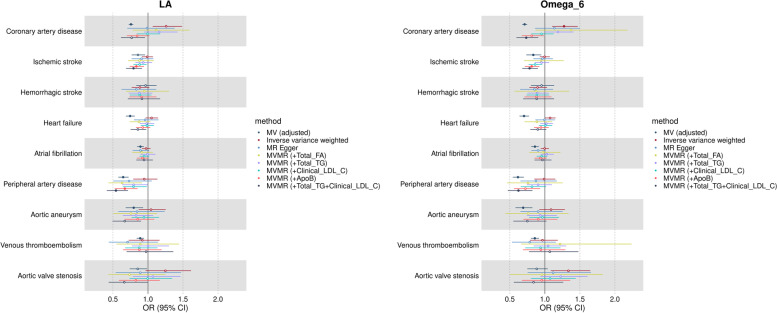
Multivariable regression and Mendelian randomization results for the risk of cardiovascular diseases associated with higher genetically predicted linoleic acid and total omega-6 fatty acids among individuals of European ancestry. Results are expressed as odds ratio of cardiovascular diseases per standard unit increase in linoleic acid and total omega-6 fatty acids with corresponding 95% confidence interval (CI). Full symbols indicate associations at *P*-value lower than the threshold accounting multiple testing (*P* < 0.006). Multivariable regression results are adjusted by for sex, age, BMI, fasting time, alcohol intake, frequency, statins use and total circulating fatty acids. MV: multivariable regression; MR: Mendelian randomization; MVMR: multivariable Mendelian randomization; FA: fatty acids; TG: triglycerides; LDL: low-density lipoprotein cholesterol; ApoB: apolipoprotein B

Higher circulating linoleic acid was related to lower risk of most cardiovascular disease endpoints. The odds ratio of disease per standard unit higher linoleic acid concentration (in models adjusted for sex, age, BMI, fasting time, alcohol intake frequency, smoking, statins use and total circulating fatty acids) was 0.76 (95% CI 0.72–0.79) for coronary artery disease, 0.86 (95% CI 0.77–0.96) for ischemic stroke, 0.97 (95% CI 0.83–1.12) for haemorrhagic stroke, 0.75 (95% CI 0.68–0.81) for heart failure, 0.89 (95% CI 0.84–0.94) for atrial fibrillation, 0.65 (95% CI 0.58–0.72) for peripheral artery disease, 0.80 (95% CI 0.68–0.93) for aortic aneurysm, 0.89 (95% CI 0.84–0.94) for venous thromboembolism and 0.85 (95% CI 0.74–0.99) for aortic valve stenosis. Similar results were observed for total omega-6 fatty acids (Fig. 3).

### Mendelian randomization analysis

#### Omega-3 PUFA (DHA and total omega-3 fatty acids)

For the pooled analyses using the univariable IVW method, the odds ratio of disease per standard unit higher genetically predicted DHA concentration was 1.12 (95% CI 0.99–1.25) for coronary artery disease, 1.06 (95% CI 0.99–1.13) for ischemic stroke, 0.93 (95% CI 0.81–1.07) for haemorrhagic stroke, 1.04 (95% CI 0.98–1.11) for heart failure, 1.04 (95% CI 0.97–1.11) for atrial fibrillation, 1.14 (95% CI 1.00–1.30) for peripheral artery disease, 1.11 (95% CI 0.95–1.30) for aortic aneurysm, 1.07 (95% CI 0.96–1.19) for venous thromboembolism, 1.36 (95% CI 1.10–1.68) for aortic valve stenosis (Fig. 2). None of these results, except for aortic valve stenosis, passed our multiple testing correction threshold (*P* < 0.006).

Overall, univariable IVW point estimates for the relation between DHA and CVDs were consistent with estimates from MR-Egger, except for peripheral artery disease and venous thromboembolism (which were slightly higher for MR-Egger), and with estimates from multivariable IVW, except for coronary artery disease and aortic aneurism (which were fully attenuated in multivariable MR adjusting for LDL-cholesterol or apolipoprotein B) (Fig. 2).

For the vast majority of exposure-outcome combinations, there was substantial heterogeneity across individual SNP estimates as indicated by Cochrane’s Q statistic *p*-values (Q *p*-value range 0.04 to 2 × 10^−102^), but limited evidence of unbalanced pleiotropy as assessed by the MR-Egger intercept test for most outcomes, except for peripheral artery disease (−0.015; *p*-value = 0.046) and venous thromboembolism (−0.018; *p*-value = 0.001) (Additional file 1: Table S10). Results were generally consistent across studies given imprecision in study-specific estimates (Additional file 2: Fig. S5A). Leave-one-out analyses indicated that main IVW results were driven by rs174528, a SNP nearby the *FADS* locus, since the removal of this SNP resulted in partial or complete attenuation of the IVW estimated effect for most outcomes (i.e. ischemic stroke, heart failure, atrial fibrillation, peripheral artery disease and aortic aneurism) and in reversal of the direction of the estimated effect for venous thromboembolism (Additional file 2: Fig. S6).

The conditional F statistics for DHA in multivariable IVW ranged from 76 to 112 (Additional file 1: Table S11). Results were similar between DHA and total omega-3, as shown in Fig. 2, Additional file 1: Table S10 and Additional file 2: Fig. S5A and 7.

#### Omega-6 PUFA (linoleic acid and total omega-6 fatty acids)

For the pooled analyses using the univariable IVW method, the odds ratio of disease per additional standard unit of genetically predicted linoleic acid concentration was 1.26 (95% CI 1.06–1.49) for coronary artery disease, 0.99 (95% CI 0.91–1.07) for ischemic stroke, 0.88 (95% CI 0.76–1.02) for haemorrhagic stroke, 1.05 (95% CI 0.96–1.14) for heart failure, 0.97 (95% CI 0.90–1.04) for atrial fibrillation, 0.95 (95% CI 0.79–1.13) for peripheral artery disease, 1.11 (95% CI 0.95–1.30) for aortic aneurysm, 0.92 (95% CI 0.72–1.17) for venous thromboembolism and 1.25 (95% CI 0.96–1.61) for aortic valve stenosis (Fig. 3). None of these results passed our multiple testing correction threshold (*P* < 0.006).

There were some inconsistencies between point estimates from univariable IVW compared to MR-Egger and multivariable IVW, though it should be noted that some estimates were very imprecise. As an example, compared to univariable IVW, estimates for the relation between circulating linoleic acid were attenuated to the null or became protective for coronary artery disease (OR 0.99; 95% CI 0.70–1.38 for MR-Egger; OR 0.77; 95% CI 0.61–0.96 for multivariable IVW simultaneously accounting for LDL-cholesterol and triglycerides) and became protective for ischemic stroke (OR 0.91; 95% CI 0.76–1.08 for MR-Egger; OR 0.79; 95% CI 0.69–0.92 for multivariable IVW accounting for triglycerides and LDL-cholesterol) and peripheral artery disease (OR 0.54; 95% CI 0.41–0.71 for multivariable IVW accounting for triglycerides and LDL-cholesterol). Overall, accounting for total fatty acids or triglycerides individually in multivariable IVW increased uncertainty (i.e. resulted in wider confidence intervals) but did not substantially change the magnitude of effect estimates as compared to univariable IVW (Fig. 3).

For vast majority of exposure-outcome combinations, there was substantial heterogeneity across individual SNP estimates as indicated by the Cochrane’s Q statistic *p*-values (Q *p*-value range 0.2 to 8 × 10^−209^), but limited evidence of unbalanced pleiotropy as assessed by the MR-Egger intercept test [intercept ranging from 0.004 (*p*-value = 0.66) to 0.023 (*p*-value = 0.13)] (Additional file 1: Table S10). Overall, leave-one-out analyses did not indicate that the main results were substantially influenced by a single SNP with the exception of rs115478735, a SNP in the *ABO* locus, for which removal increased the magnitude and precision for the estimated effect of linoleic acid on venous thromboembolism risk (Additional file 2: Fig. S8).

The conditional F statistics for linoleic acid in multivariable IVW ranged from 52 to 128 (Additional file 1: Table S11). Results were generally consistent across studies given imprecision in study-specific estimates, except for haemorrhagic stroke and heart failure (Additional file 2: Fig. S5B), and between linoleic acid and total omega-6 (Fig. 3, Additional file 1: Table S10 and Additional file 2: Fig. S5B and S9).

### Positive exposure control

Our positive control exposure analyses confirmed that higher circulating LDL-cholesterol and apolipoprotein B were related to higher risk of several cardiovascular disease outcomes. As an example, the odds ratio of disease per additional standard unit of genetically predicted LDL-cholesterol concentration was 1.55 (95% CI 1.43–1.68) for coronary artery disease, 1.13 (95% CI 1.05–1.23) for ischemic stroke, 0.91 (95% CI 0.81–1.02) for haemorrhagic stroke, 1.13 (95% CI 1.06–1.22) for heart failure, 1.04 (95% CI 0.98–1.11) for atrial fibrillation, 1.25 (95% CI 1.08–1.45) for peripheral artery disease, 1.17 (95% CI 1.02–1.35) for aortic aneurysm, 0.99 (95% CI 0.80–1.21) for venous thromboembolism and 1.35 (95% CI 1.13–1.61) for aortic valve stenosis (Additional file 2: Fig. S10). Removing a SNP within the *FADS* locus (i.e. rs102275) did not alter results from the positive control analyses (Additional file 1: Table S12).

## Discussion

Using the largest-scale genetic association data available for fatty acids from over 114,000 UK Biobank participants, we identified a much larger number of genetic variants strongly and independently associated with circulating PUFA concentration compared to previous GWAS [8, 9, 11–13], which enabled us to conduct key sensitivity analyses such as MR-Egger and multivariable MR, both of which preferably require a large number of independent instruments. As expected, many of these genetic variants mapped to genes involved in lipoprotein-related metabolism. This poses a challenge to Mendelian randomization studies of fatty acids since using SNPs from a single gene region involved in fatty acid metabolism (e.g. *FADS*) is likely to be more specific but not amenable to most pleiotropy-robust Mendelian randomization methods, while using SNPs across the genome is likely to introduce non-specificity but allow the use of pleiotropy-robust methods.

When using these multiple genetic variants as instruments for Mendelian randomization, our findings did not confirm the inverse association observed in conventional multivariable regression and provided weak evidence of higher genetically predicted DHA (and total omega-3 fatty acids) concentration being related to higher risk of some cardiovascular endpoints. However, overall, Mendelian randomization findings did not pass our criteria for multiple testing correction and were attenuated when accounting for LDL-cholesterol/apolipoprotein B or excluding a SNP in the vicinity of the *FADS* locus. Mendelian randomization findings for higher genetically predicted linoleic acid (and total omega-6) concentration were inconsistent across different cardiovascular endpoints and methods and did not confirm the inverse association observed in conventional multivariable regression. There was weak evidence of higher genetically predicted linoleic acid being related to lower risk of ischemic stroke and peripheral artery disease after accounting for LDL-cholesterol/apolipoprotein B. Despite the large increase in the number of instruments in our analyses, there remains considerable imprecision in estimates for the effect of circulating fatty acids on the risk of some cardiovascular disease outcomes. As an example, IVW estimates for the relation of DHA with the risk of aortic aneurysm was 1.11 (95% CI 0.95–1.30), while IVW estimates for the relation of our positive control exposure (LDL-cholesterol) with the same outcome was 1.17 (95% CI 1.02–1.35). This indicates that we cannot confidently rule out the presence of clinically meaningful effects due to the considerable uncertainty in some results.

Previous metanalyses of classical observational studies indicate that higher circulating long-chain omega-3 and omega-6 PUFA are either not associated or associated with lower risk of coronary artery disease and stroke [61–64]. Cochrane recently published a series of systematic reviews of RCTs with the overall conclusion that increasing omega-3, omega-6 or total PUFA intake, via supplementation or diet, has modest to no effect on CVD events or mortality [4–6]. Since then, further large-scale RCTs on long-chain omega-3 PUFA have been published and yielded conflicting results [65–67]. In addition, most Mendelian randomization studies, as well as classical observational studies and RCTs, have focussed on exploring the effect of PUFA on the risk of coronary artery disease and, to a lesser extent, ischemic stroke. The effects of these fatty acids on other types of cardiovascular endpoints, such as heart failure and atrial fibrillation, have been under explored.

Integrating multiple lines of evidence to resolve controversies in research on cardiovascular health effects of fatty acids is essential. However, directly comparing results from different study designs in this context is not straightforward. For illustration, in intervention studies, the effect of PUFA supplementation or diet intake on CVD endpoints is frequently tested over relatively short periods of time due to logistical issues [4–6] and, in some instances, may depend on the overall diet composition [3]. On the other hand, genetic proxies of circulating PUFA affect their metabolism (not intake) are assumed to have lifelong effects and have pleiotropic effects on lipoprotein-related traits.

Mendelian randomization can provide a valid test of the presence of a causal effect if genetic variants are relevant and valid instruments for the exposure of interest.

Regarding instrument relevance, we have selected independent SNPs strongly associated with circulating PUFA concentration, which explained from 4.8 to 7.9% of phenotypic variance (mean F statistics 109–201) among the UK Biobank participants (discovery sample). In addition, we replicated these associations in an independent dataset [12] using the same NMR metabolomics platform as the one used in the UK Biobank participants (median sample size 13,516), where SNPs explained 3.1 to 6.9% of phenotypic variance in circulating PUFA. This indicates that bias due to weak instruments is unlikely to be substantial in our analyses, even though bias due to winner’s curse (due to using the UK Biobank to select SNPs and estimate their effect on PUFA) could affect the magnitude of effect estimates. There was little evidence that the selected SNPs impacted PUFA composition in independent datasets with more detailed data on individual PUFA, particularly after removing SNPs nearby the *FADS* locus. This might be related to differences in assays, units of analyses or statistical power.

Instrument validity requires that any effect from genetic instrument to the outcome is completely mediated by the exposure of interest. This assumption could be violated in several scenarios, such as in the presence of confounding due to population stratification or horizontal pleiotropy.

Bias by population stratification could result if heterogeneity in genetic ancestry in a given sample was related to different distributions of genetic instrument and outcome. To mitigate that, heterogeneity in genetic ancestry was accounted for when generating genetic association data for cardiovascular outcomes in all data sources by correcting for genomic inflation factor, adjusting for principal components of ancestry or using mixed linear models as detailed in Table S2 (Additional file 1). In addition, the overall consistency of findings across multiple studies provides some reassurance against findings being explained by population stratification.

Horizontal pleiotropy is one of the major threats to the validity of Mendelian randomization studies. By conducting a series of analyses for functional mapping and annotation of fatty acid genetic association results, we showed that genetic variants associated with fatty acids are strongly enriched for genes and pathways involved in lipoprotein metabolism, particularly (triglyceride-rich or high-density) lipoprotein particle remodelling, apolipoprotein binding and reverse cholesterol transport. For illustration, some PUFA SNPs mapped to genes encoding proteins targeted by lipid-lowering drugs, such as *HMGCR* (Entrez Gene 3156), *PCSK9* (Entrez Gene 255738) and *CETP* (Entrez Gene 1071) [68, 69]. Considering the pivotal role of lipoprotein metabolism in the aetiology of several cardiovascular diseases, this stresses that the assumption of no horizontal pleiotropy (or the weaker versions of this assumption by MR-Egger) in our analyses is likely implausible. We tried to mitigate that by using multivariable MR to account for total fatty acids, given the lack of specificity of the selected instruments for specific fatty acids, and for triglycerides, LDL-cholesterol or apolipoprotein B, which are key determinants of several CVDs reflecting lipoprotein metabolism. Accounting for LDL-cholesterol/apolipoprotein B revealed a potential direct protective effect of linoleic acid on the risk of ischemic stroke and peripheral artery disease, suggesting that horizontal pleiotropy via LDL-cholesterol might have masked some true underlying protective effect of linoleic acid. In addition, Mendelian randomization findings for the relation between DHA and several CVDs were attenuated when excluding SNPs in the vicinity of the *FADS* locus. On the one hand, SNPs regulating *FADS1* and *FADS2* are expected to be more credible instruments given their proximal relation with PUFA biosynthesis. However, these SNPs have been shown to be associated with numerous fatty acids and non-fatty acid traits and may not be valid instruments for circulating DHA [22]. Results from multivariable regression were often inconsistent with results from MR IVW (e.g. multivariable regression and IVW estimates were in opposite direction for the association between PUFA and CAD risk). While the reason for that is not entirely clear, this is possibly related to pleiotropic genetic instruments since accounting for LDL-cholesterol in MR analyses attenuated those differences.

## Conclusions

Overall, our Mendelian randomization findings do not support a protective role of circulating DHA, total omega-3, linoleic acid and total omega-6 concentration on the risk of CVDs, as observed in conventional analysis. However, horizontal pleiotropy via lipoprotein-related traits could be a key source of bias in our analyses despite our attempts to account for that.

## Supplementary Information

**Additional file 1: Supplementary Tables S1–S11**.

**Additional file 2: Supplementary Figures S1–S10**.

**Additional file 3.**

## Data Availability

The genetic association data on fatty acids and other NMR metabolic traits generated by this study were deposited at the IEU Open GWAS Project [47, 48]. Genetic association data from GWAS metanalyses and FinnGen for cardiovascular endpoints from contributing studies is publicly available as detailed in Additional file 1: Table S2 [39–43]. Researchers can apply for access to the UK Biobank data via the Access management System (AMS) (https://www.ukbiobank.ac.uk/enable-your-research/apply-for-access). The code for the MR analyses is available at [https://github.com/mcarolborges/NMR_PUFA.git].

## Abbreviations

ALA: Alpha-linolenic acid
BMI: Body mass index
CARDIoGRAMplusC4D: Coronary Artery Disease Genome-Wide Replication and Meta-analysis Plus the Coronary Artery Disease Genetics Consortium
CVDs: Cardiovascular diseases
D5D: Delta-5 desaturase
D6D: Delta-6 desaturase
DHA: Docosahexaenoic acid
eQTL: Expression quantitative trait loci
FUMA GWAS: Functional Mapping and Annotation of Genome-Wide Association Studies
GC: Gas chromatography
GWAS: Genome-wide association study
HERMES: The Heart Failure Molecular Epidemiology for Therapeutic Targets
INSIDE assumption: Instrument Strength Independent of Direct Effects assumption
IVW method: Inverse variance weighted method
LA: Linoleic acid
LD: Linkage disequilibrium
LDL: Low-density lipoprotein
LDSC: LD score regression
LMM: Linear mixed model
MAF: Minor allele frequency
MS: Mass spectrometry
NMR: Nuclear magnetic resonance
PUFA: Polyunsaturated fatty acids
QC: Quality control
RCTs: Randomized controlled trials
SNPs: Single-nucleotide polymorphisms
