# Supplementary material for: Role of circulating polyunsaturated fatty acids on cardiovascular diseases risk: analysis using Mendelian randomization and fatty acid genetic association data from over 114,000 UK Biobank participants
Source: BMC Med. 2022 Jun 13;20:210. doi: 10.1186/s12916-022-02399-w (PMC9190170; doi:10.1186/s12916-022-02399-w)

**Supplementary figure 1**. Functional annotation of genetic variants associated with fatty acids measures using FUMA

FUMA identifies as *independent significant SNPs* those associated with the trait of interested at p-value < 5e-8 in the GWAS summary data and not in strong LD with each other (R^2^ < 0.6 using EUR 1000G phase3 as reference panel). For each independent significant SNP, all known SNPs with MAF ≥ 1% in strong LD (R^2^ ≥ 0.6), either present in the GWAS summary data and/or reference panel, are included for further annotation (i.e. *candidate SNPs*). *Independent lead SNPs* are a subset of independent significant SNPs in weak LD (R^2^ < 0.1). Additionally, if LD blocks of independent significant SNPs are closely located to each other (< 250 kb based on the most right and left SNPs from each LD block), they are merged into one *genomic risk locus*. Therefore, each genomic locus can contain multiple independent significant SNPs and lead SNPs. SNPs are mapped to genes based on positional, eQTL and chromatin interaction information of SNPs. Functional consequences of SNPs on genes are obtained by performing ANNOVAR (“gene-based annotation”) using Ensembl genes (build 85). SNPs can be annotated to more than one gene in case of intergenic SNPs which are annotated to the two closest up- and down-stream genes. FUMA: Functional Mapping and Annotation of Genome-wide Association Studies; SNPs: single nucleotide polymorphisms; GWAS: genome-wide association studies; LD: *linkage disequilibrium*; MAF: minor allele frequency.

**Supplementary figure 2**. Variant-based Manhattan plot of genetic association results for fatty acids measures

**Supplementary figure 3**. Quantile-quantile (QQ) plot of genetic association results for fatty acids measures

**Supplementary figure 4**. Impact of genetically-predicted total omega-3 and total omega-6 on the circulating PUFA composition among individuals of European ancestry before and after excluding SNPs nearby the *FADS* locus.

Results are expressed as z-statistics (effect estimate / standard error) for the variation in individual omega-3 and omega-6 fatty acids (y-axis) across multiple data sources (x-axis) per unit increase in genetically-predicted total omega-3 and total omega-6. Blue, red, and grey boxes denote, respectively, decreases, increases, and no change in individual PUFA, while white boxes represent missing data. Asterisks indicate P value: < $5\times{10}^{-8}$ (***), < $5\times{10}^{-5}$ (**), and < $5\times{10}^{-2}$ (*). Plasma FA: plasma fatty acids; RBC FA: red blood cell membrane fatty acids; N: median sample size used for estimating SNP-fatty acids association; GC: gas chromatography; NMR: nuclear magnetic resonance; MS: mass spectrometry.

**Supplementary figure 5**. Study-specific Mendelian randomization results for the risk of cardiovascular diseases among individuals of European ancestry associated with (A) higher genetically-predicted DHA and total omega-3, and (B) higher genetically-predicted linoleic acid and total omega-6 fatty acids.

Results are estimated using the inverse variance weighted method and expressed as odds ratio of cardiovascular diseases per standard unit increase in DHA, total omega-3, linoleic acid, and total omega-6 fatty acids. Full symbols indicate associations at P-value lower than the threshold accounting multiple testing (P < 0.006). DHA: docosahexaenoic acid.

**Supplementary figure 6**. Leave-one-out Mendelian randomization analyses for the relation between genetically-predicted DHA and the risk of cardiovascular diseases among individuals of European ancestry

Results are expressed as log odds ratio of cardiovascular diseases per standard unit increase in DHA. DHA: docosahexaenoic acid.

**Supplementary figure 7**. Leave-one-out Mendelian randomization analyses for the relation between genetically-predicted total omega-3 fatty acids and the risk of cardiovascular diseases among individuals of European ancestry

Results are expressed as log odds ratio of cardiovascular diseases per standard unit increase in total omega-3 fatty acids.

**Supplementary figure 8**. Leave-one-out Mendelian randomization analyses for the relation between genetically-predicted linoleic acid and the risk of cardiovascular diseases among individuals of European ancestry

Results are expressed as log odds ratio of cardiovascular diseases per standard unit increase in linoleic acid.

**Supplementary figure 9**. Leave-one-out Mendelian randomization analyses for the relation between genetically-predicted total omega-6 fatty acids and the risk of cardiovascular diseases among individuals of European ancestry

Results are expressed as log odds ratio of cardiovascular diseases per standard unit increase in total omega-6 fatty acids.

**Supplementary figure 10**. Positive exposure control Mendelian randomization analysis estimating the relation between genetically-predicted LDL-cholesterol and apolipoprotein B on the risk of cardiovascular diseases among individuals of European ancestry.

Results are expressed as odds ratio of cardiovascular diseases per standard unit increase in LDL-cholesterol and apolipoprotein B. Full symbols indicate associations at P-value lower than the threshold accounting multiple testing (P < 0.006).

**Supplementary figure 1**

**
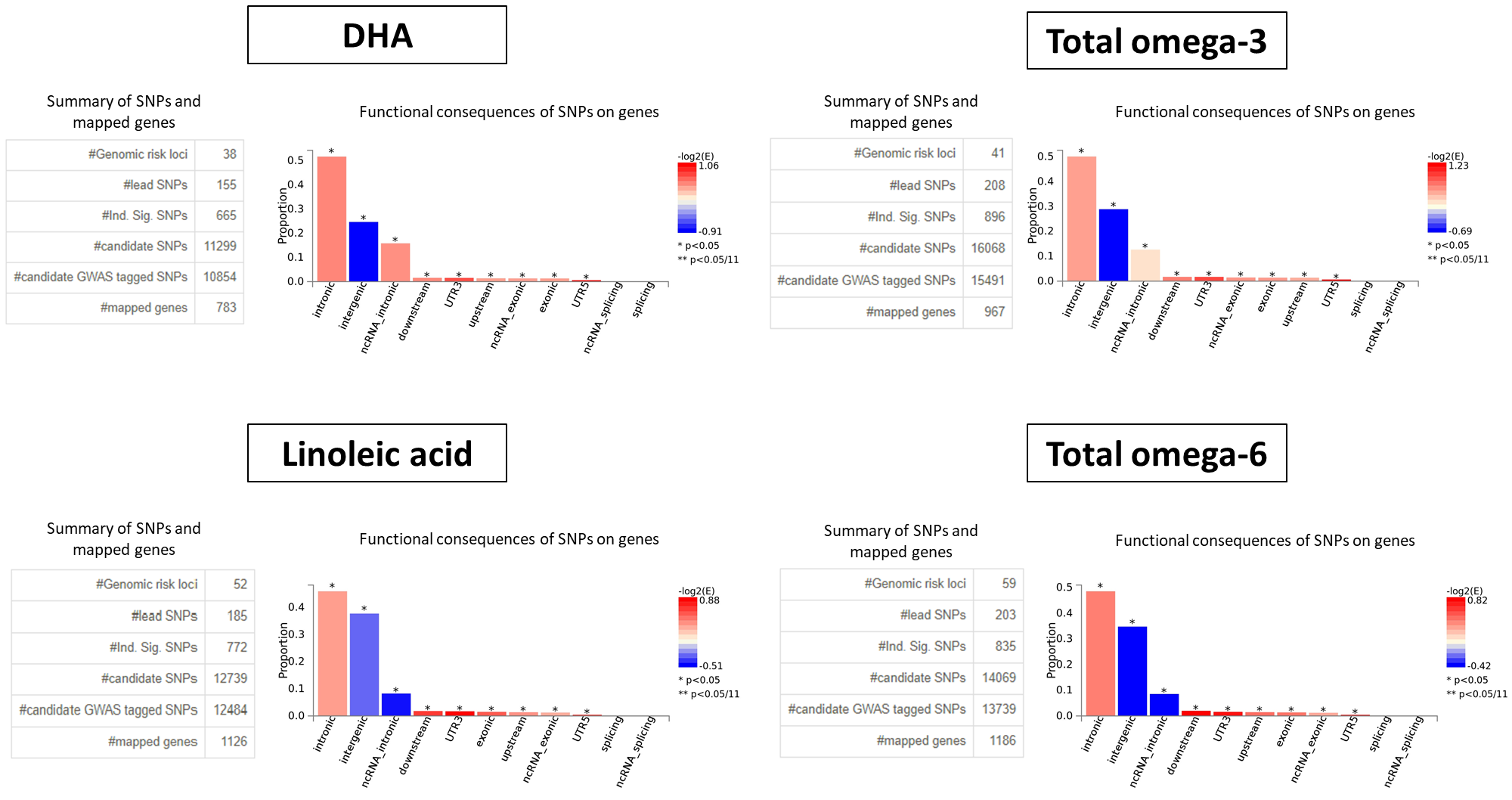
**

**Supplementary figure 2**

**
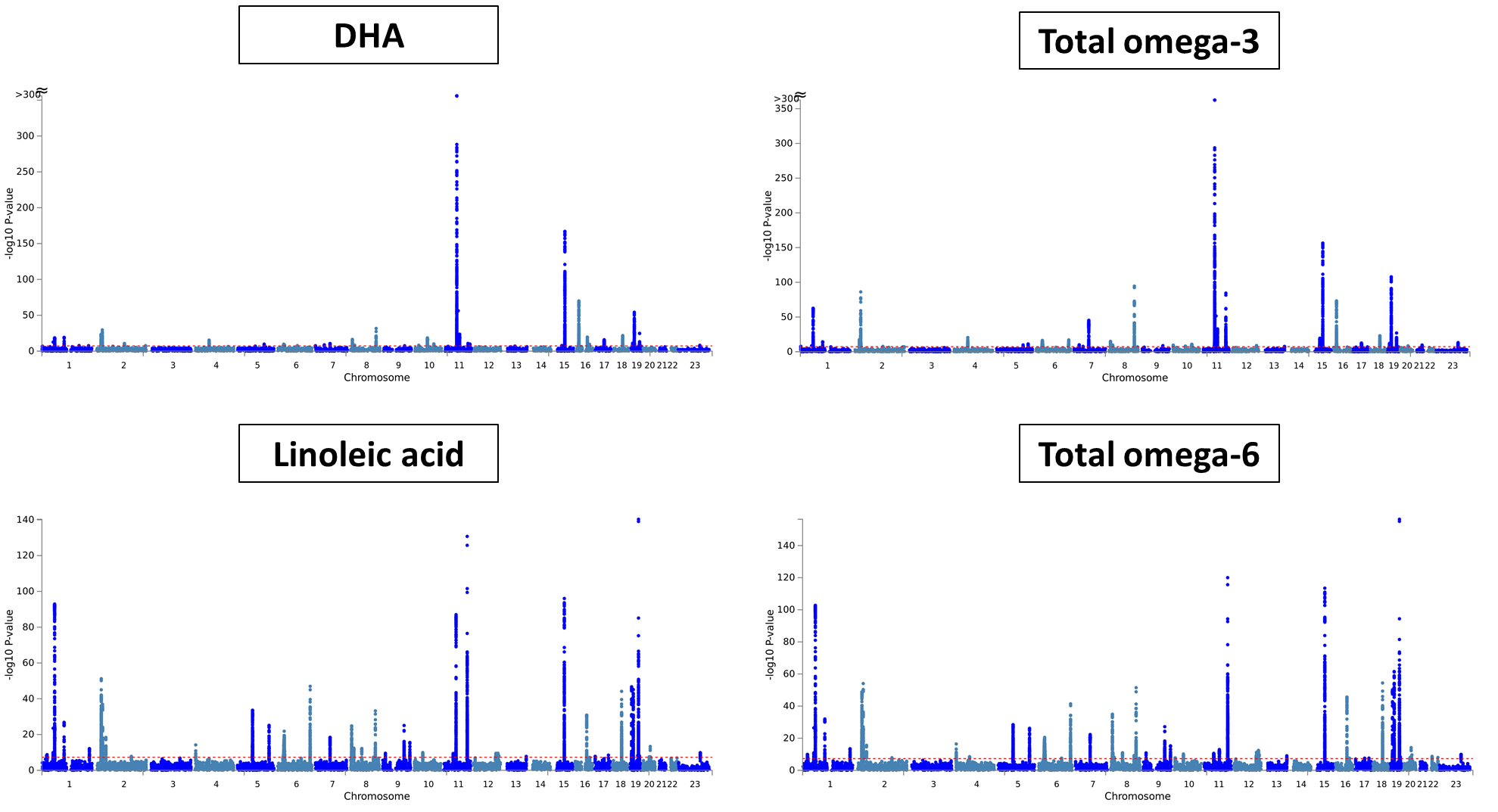
**

**Supplementary figure 3**


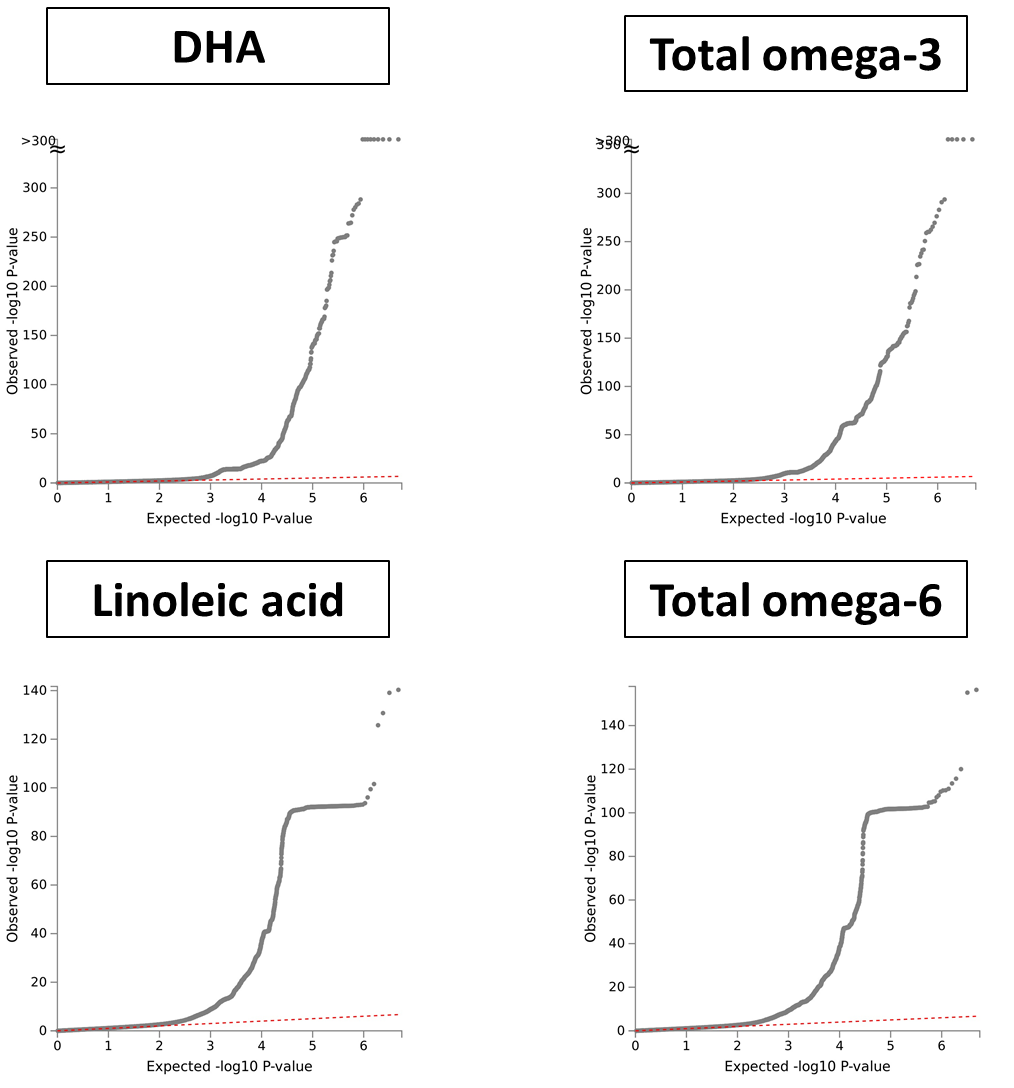


**Supplementary figure 4**


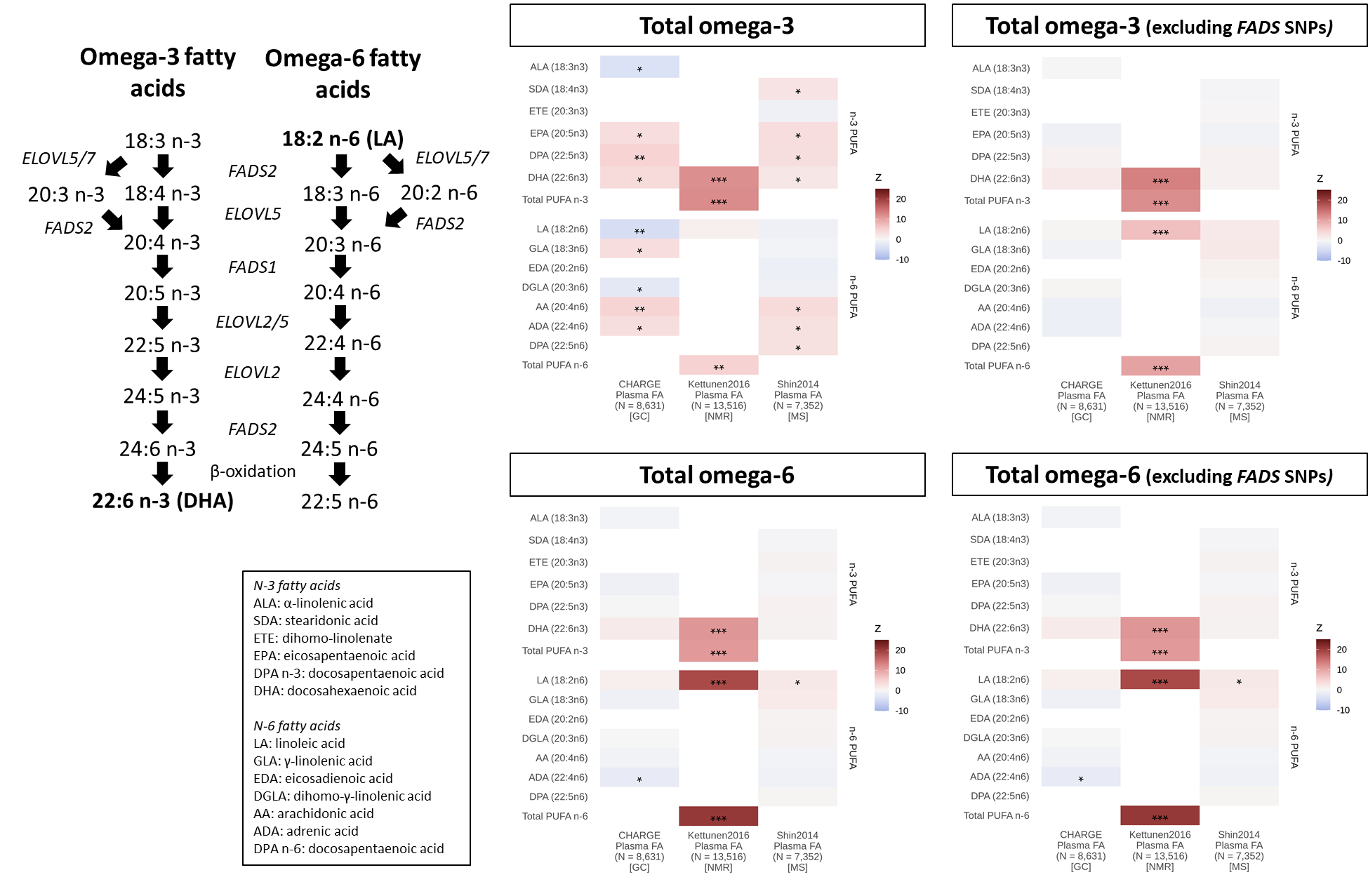


**Supplementary figure 5A**


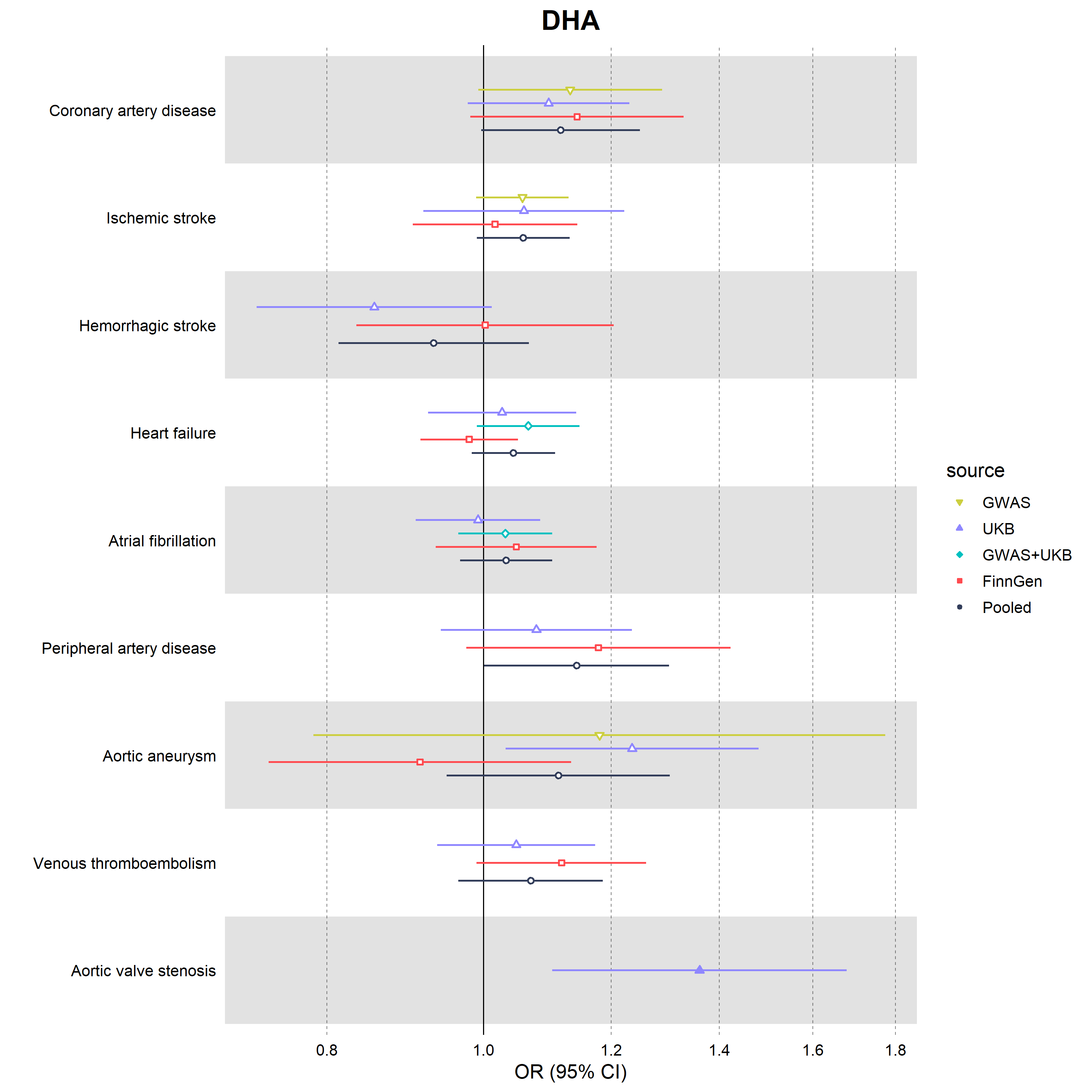

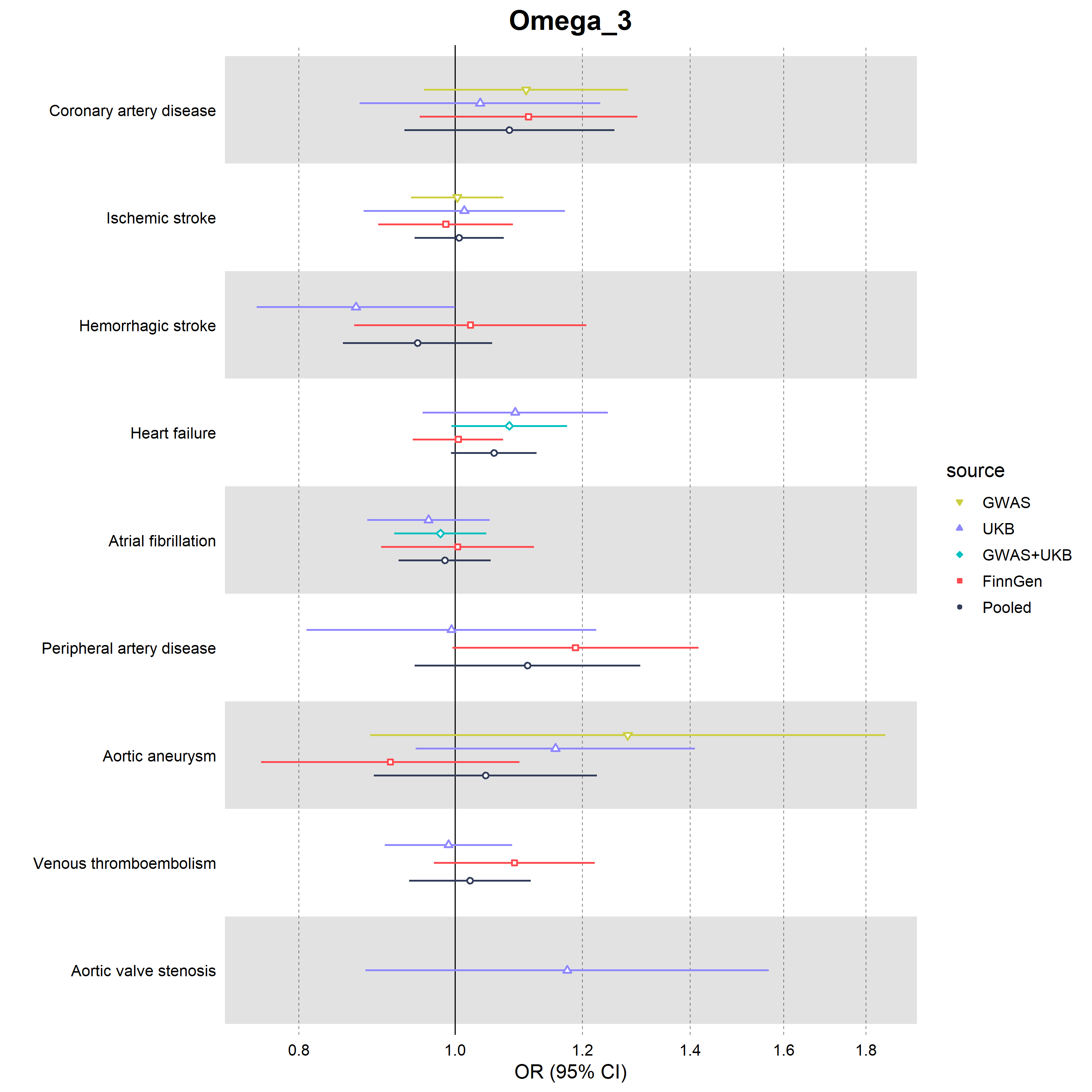


**Supplementary figure 5B**


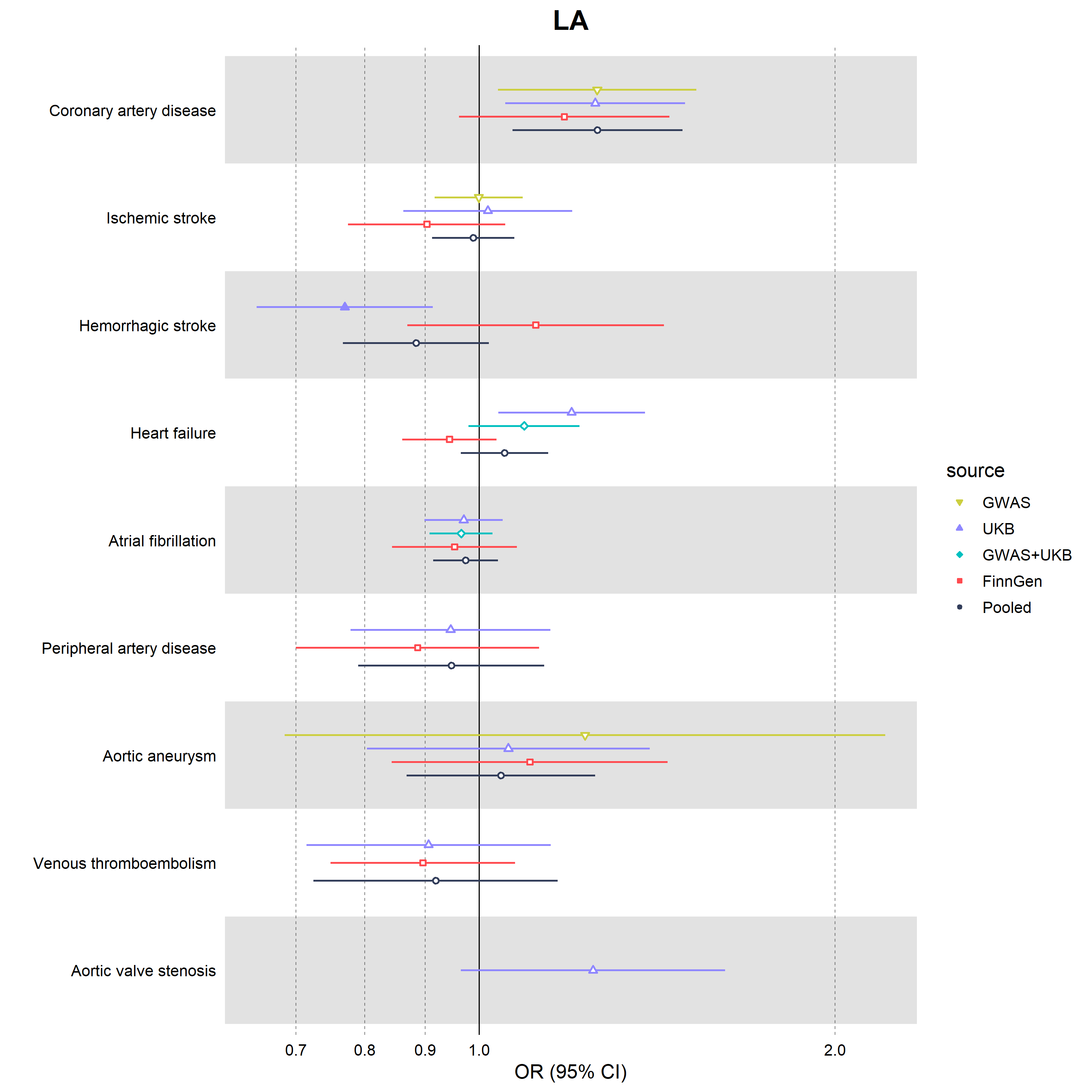

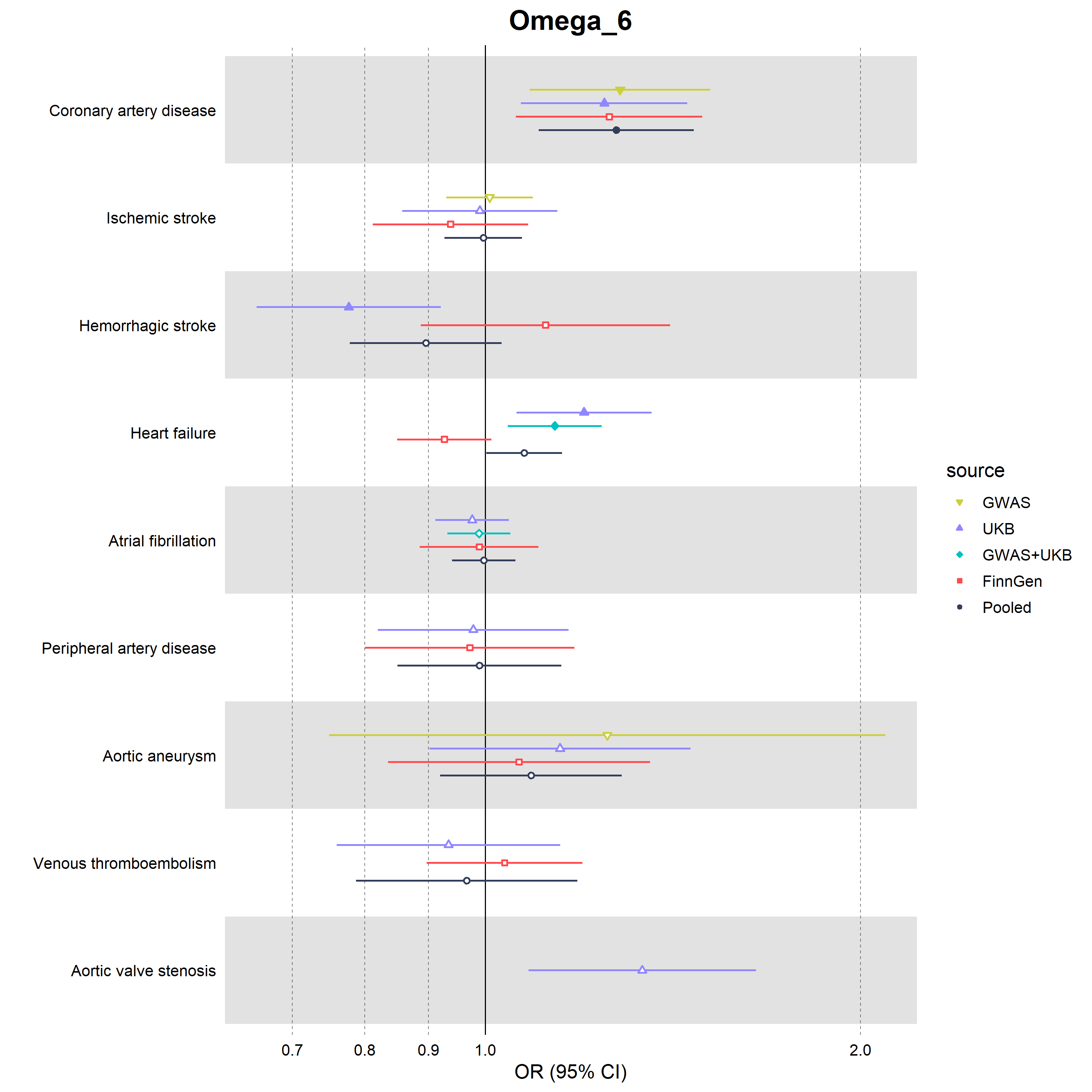


**Supplementary figure 6**

**
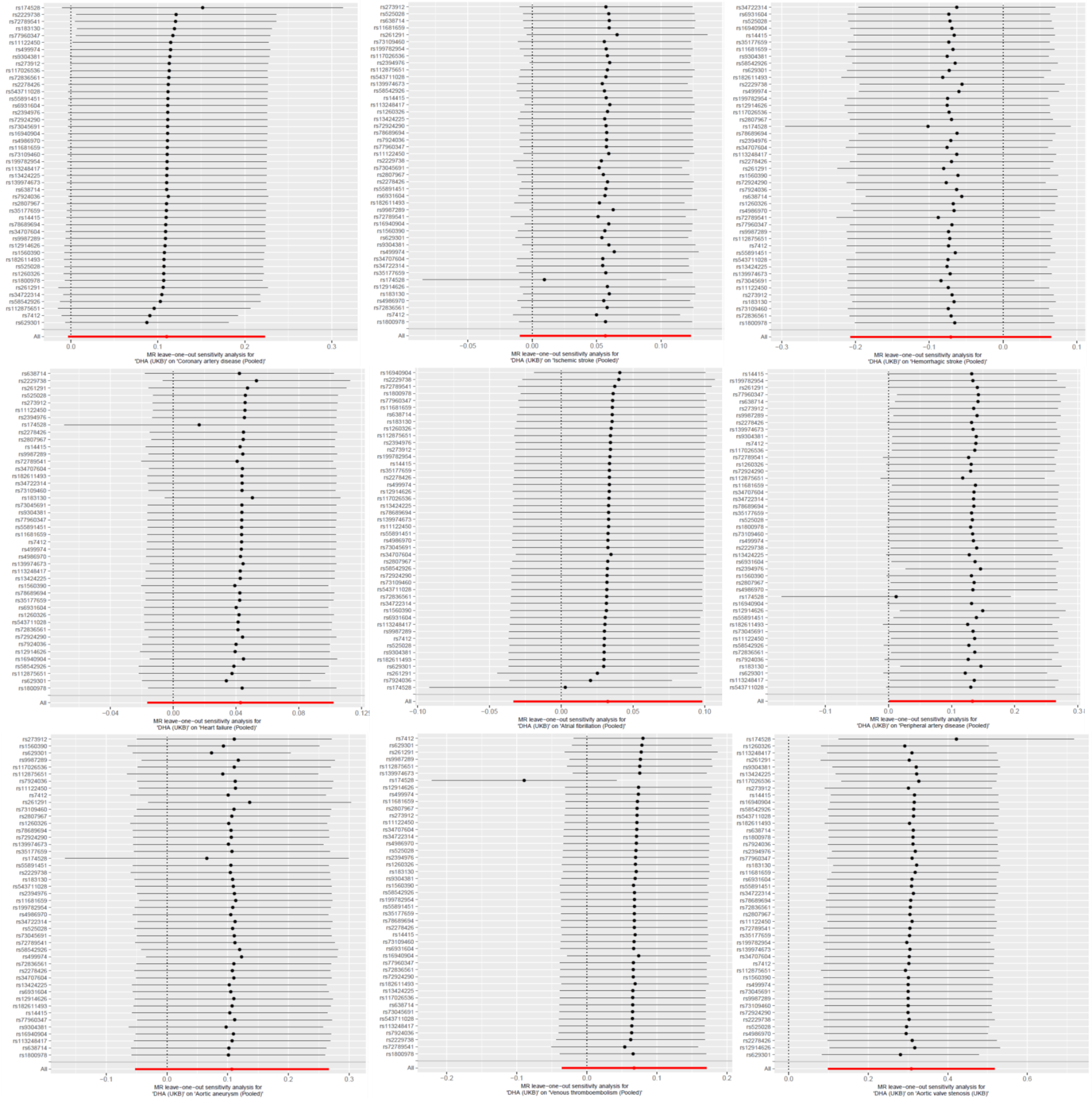
**

**Supplementary figure 7**

**
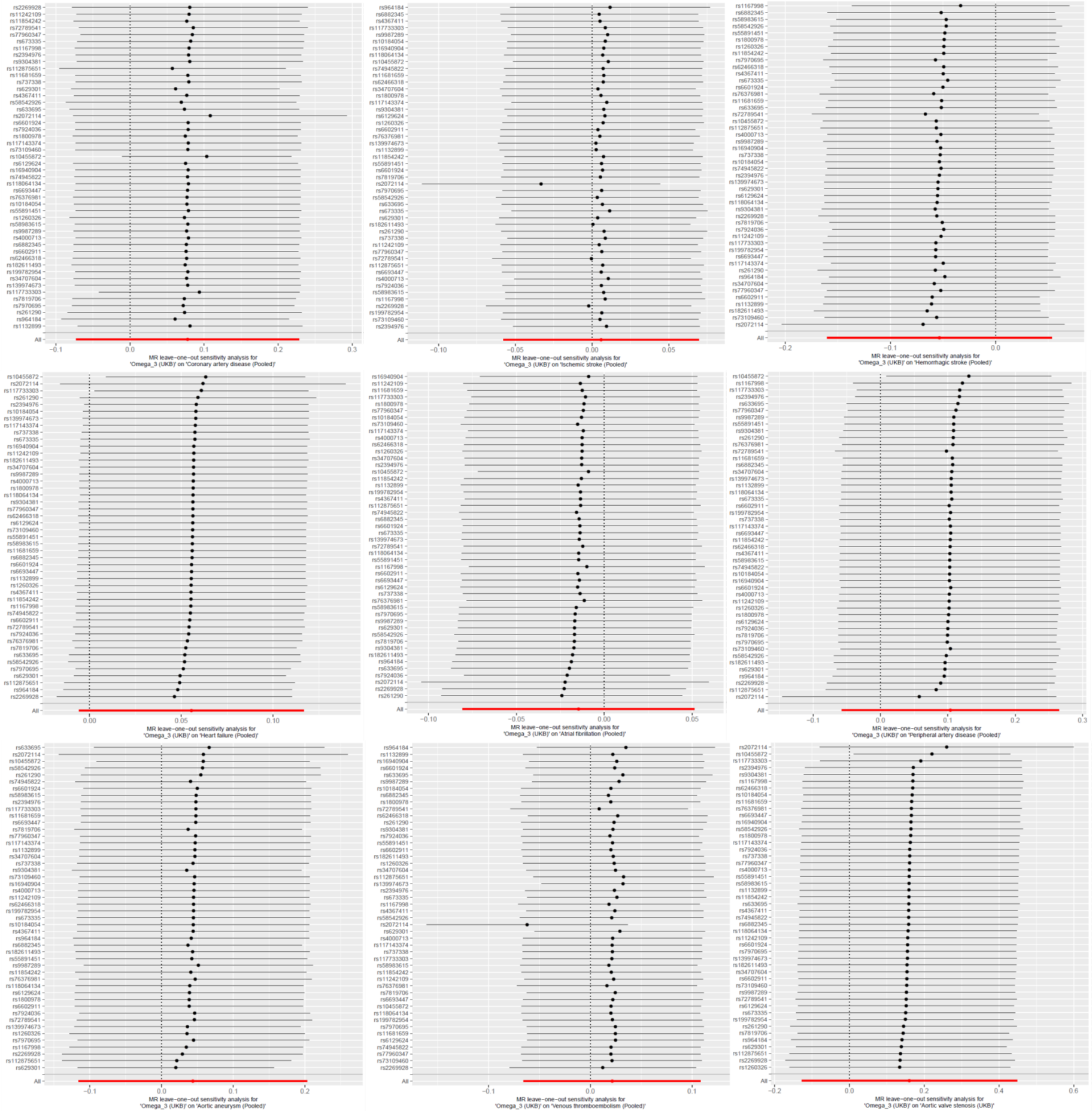
**

**Supplementary figure 8**

**
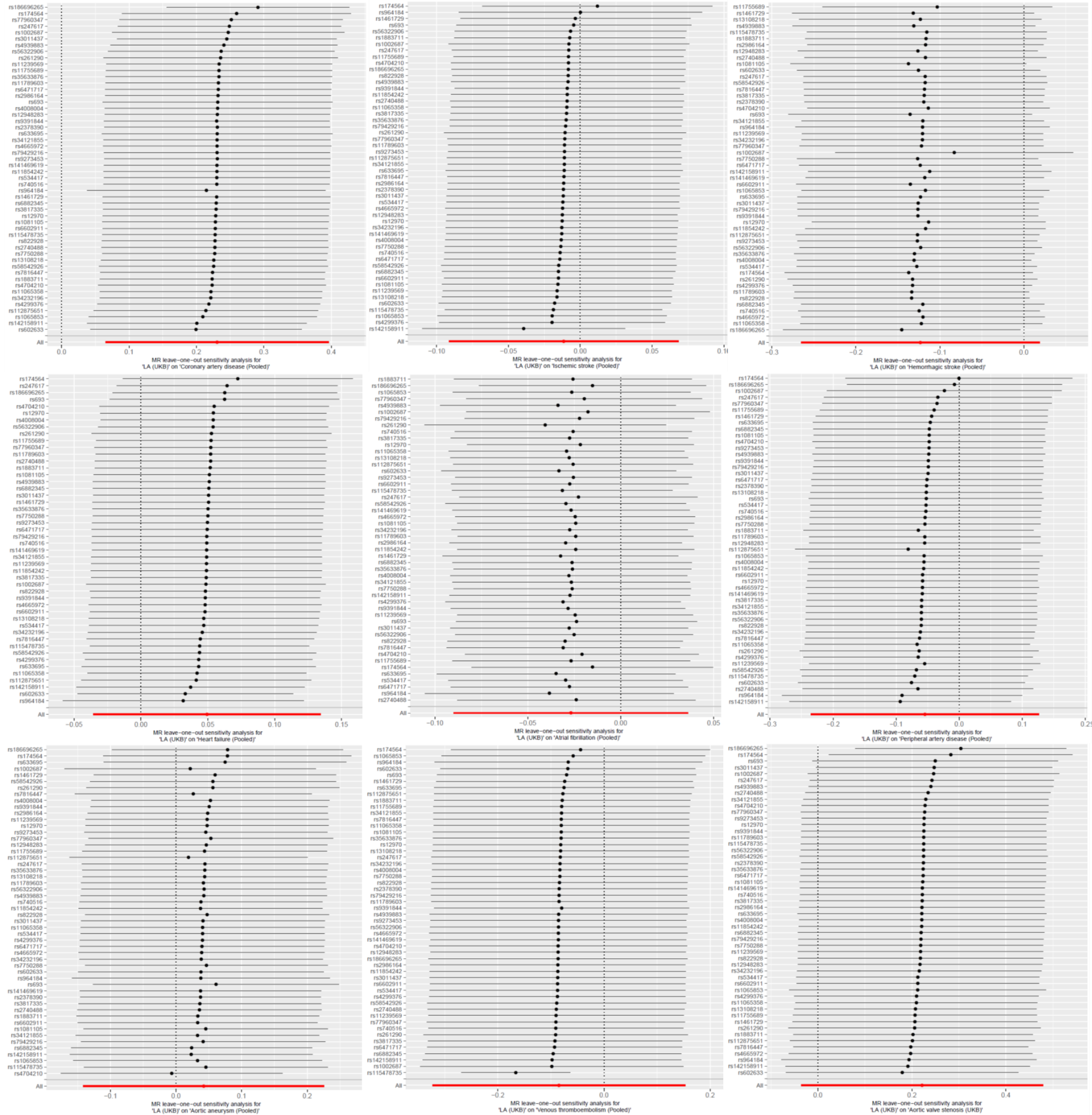
**

**Supplementary figure 9**

**
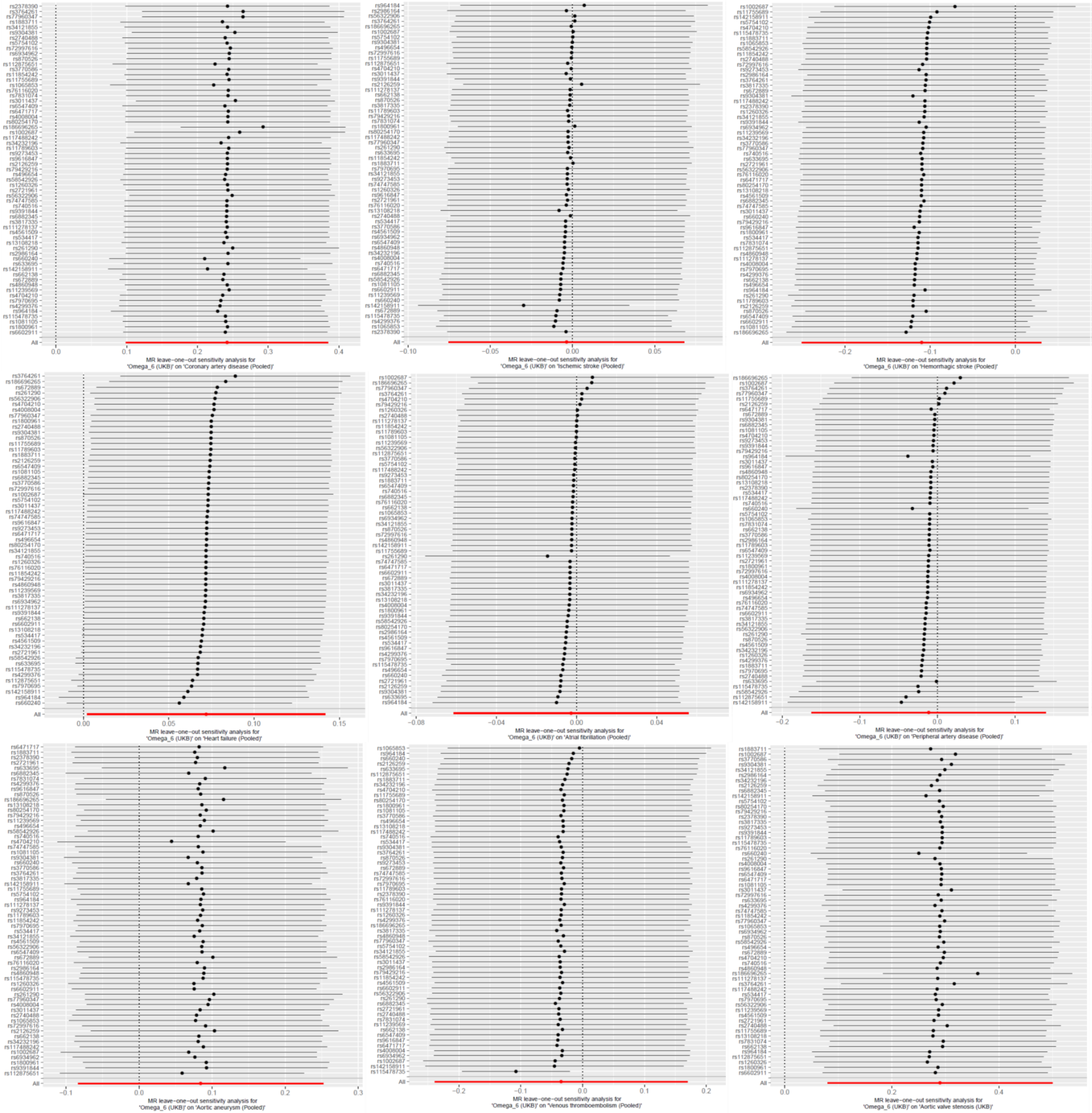
**

**Supplementary figure 10**


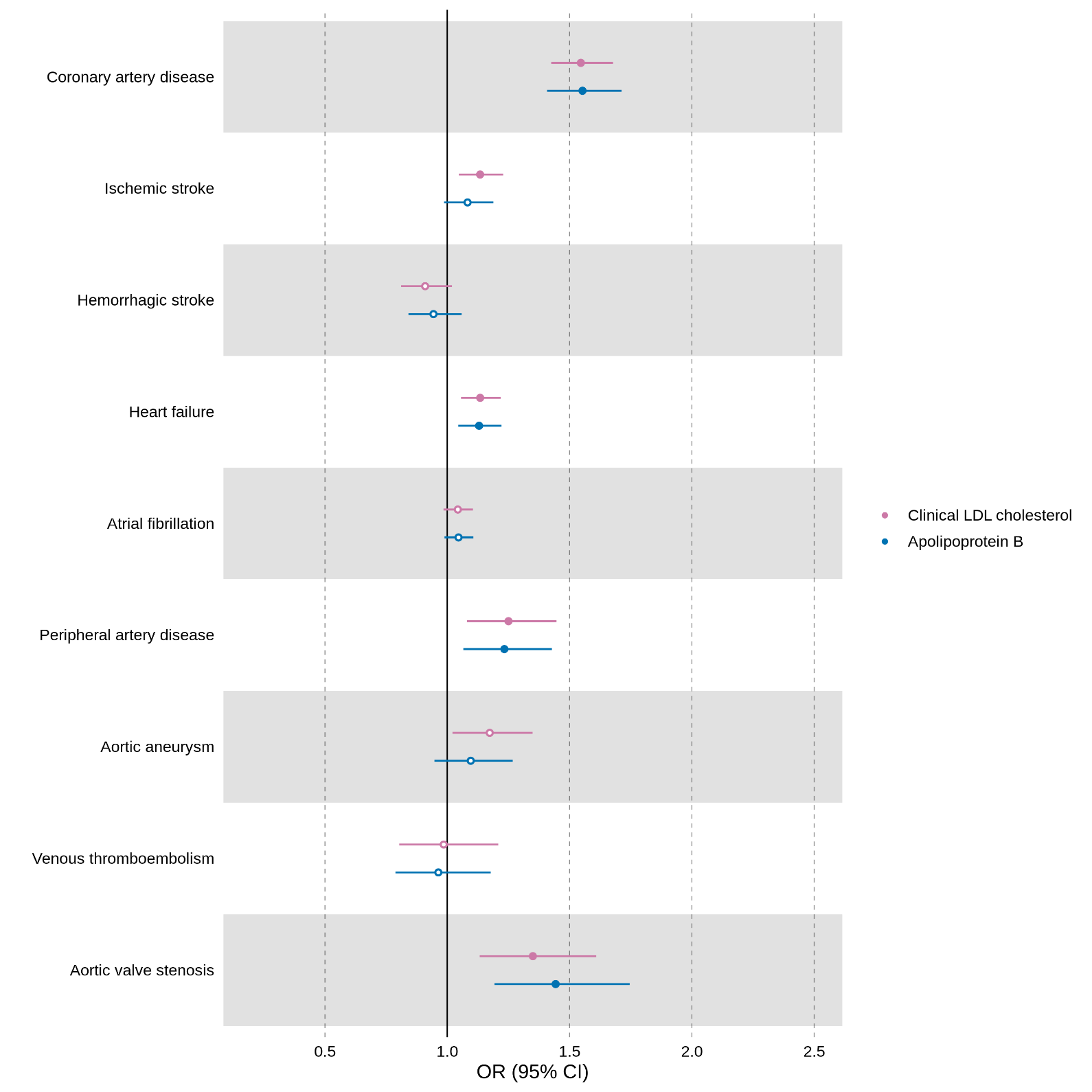

Supplement: Supplementary file 2 — Additional file 2: Supplementary Figures S1–S10. [file 12916_2022_2399_MOESM2_ESM.docx]
